# Alterations in the coupling functions between cerebral oxyhaemoglobin and arterial blood pressure signals in post-stroke subjects

**DOI:** 10.1371/journal.pone.0195936

**Published:** 2018-04-18

**Authors:** Honglun Su, Congcong Huo, Bitian Wang, Wenhao Li, Gongcheng Xu, Qianying Liu, Zengyong Li

**Affiliations:** 1 Key Laboratory for Biomechanics and Mechanobiology of Ministry of Education, School of Biological Science and Medical Engineering, Beihang University, Beijing, P.R. China; 2 School of Mechanical Engineering, Shandong University, Jinan, P.R. China; 3 Beijing Key Laboratory of Rehabilitation Technical Aids for Old-Age Disability, National Research Center for Rehabilitation Technical Aids, Beijing, P. R. China; 4 Key Laboratory of Rehabilitation Aids Technology and System of the Ministry of Civil Affairs, Beijing, P. R. China; Henry Ford Health System, UNITED STATES

## Abstract

Cerebral autoregulation (CA) is the complex homeostatic regulatory relationship between blood pressure (BP) and cerebral blood flow (CBF). This study aimed to analyze the frequency-specific coupling function between cerebral oxyhemoglobin concentrations (delta [HbO_2_]) and mean arterial pressure (MAP) signals based on a model of coupled phase oscillators and dynamical Bayesian inference. Delta [HbO_2_] was measured by 24-channel near-infrared spectroscopy (NIRS) and arterial BP signals were obtained by simultaneous resting-state measurements in patients with stroke, that is, 9 with left hemiparesis (L–H group), 8 with right hemiparesis (R–H group), and 17 age-matched healthy individuals as control (healthy group). The coupling functions from MAP to delta [HbO_2_] oscillators were identified and analyzed in four frequency intervals (I, 0.6–2 Hz; II, 0.145–0.6 Hz; III, 0.052–0.145 Hz; and IV, 0.021–0.052 Hz). In L–H group, the CS from MAP to delta [HbO_2_] in interval III in channel 8 was significantly higher than that in healthy group (*p* = 0.003). Compared with the healthy controls, the coupling in MAP→delta [HbO_2_] showed higher amplitude in interval I and IV in patients with stroke. The increased CS and coupling amplitude may be an evidence of impairment in CA, thereby confirming the presence of impaired CA in patients with stroke. In interval III, the CS in L–H group from MAP to delta [HbO_2_] in channel 16 (*p* = 0.001) was significantly lower than that in healthy controls, which might indicate the compensatory mechanism in CA of the unaffected side in patients with stroke. No significant difference in region-wise CS between affected and unaffected sides was observed in stroke groups, indicating an evidence of globally impaired CA. These findings provide a method for the assessment of CA and will contribute to the development of therapeutic interventions in stroke patients.

## Introduction

The brain has a high metabolic demand, thereby requiring adequate and timely nutrient and oxygen supply. Cerebral autoregulation (CA) is the complex homeostatic regulatory relationship between blood pressure (BP) and cerebral blood flow (CBF)[1–3]. It describes the ability of the brain to maintain a relatively constant CBF when faced with perturbations in arterial BP (ABP) through complex myogenic, neurogenic, and metabolic mechanisms which can protect the cerebral parenchyma from hyper- or hypo-perfusion injuries[4]. Autoregulation dysfunction accelerates the neurological disorders, such as stroke, brain lesions, and infections of the central nervous system[5].

The CA is significantly impaired in patients with stroke compared with controls, even after minor stroke[6–8]. Elderly patients with impaired CA may be at risk of brain damage from minor decrease in BP. These results may cause secondary injury in patients with stroke. Thus, monitoring and controlling the autoregulation capacity through hemodynamic management strategies in patients with stroke are important. With the transcranial Doppler (TCD) and continuous BP measurement techniques, most studies have assessed its autoregulatory capacity based on correlation coefficient between the cerebral perfusion pressure and CBF velocity[6, 9, 10]. These studies have demonstrated that high correlation coefficient between the cerebral perfusion pressure and CBF velocity indicates pressure-dependent flow and impaired autoregulation, whereas a low correlation indicates intact autoregulation[10]. However, at present, the functional mechanism and the causality underlying the coupling interaction between CBF and ABP have not been fully studied.

Near-infrared spectroscopy (NIRS) technique can noninvasively and continuously measure cerebral changes in the local oxygenated and deoxygenated hemoglobin concentrations on the cerebral cortex[11, 12].With special sensitivity to the microvasculature, the NIRS can provide a surrogate of fluctuations in CBF to monitor dynamic CA[13, 14]. NIRS has several potential advantages compared with TCD in terms of clinical application. For example, NIRS is relatively insensitive to movement artifacts and is portable, enabling long term monitoring of the hemodynamic activity and requiring minimal caregiver manipulation in patients with stroke at bedside[15]. In addition, NIRS-based measures of CA can provide information on specific areas with focal dysautoregulation[16].

Spontaneous oscillations are generally found in the spectral analysis of changes in the cerebral oxyhemoglobin concentration (delta [HbO_2_]) signals measured by NIRS in the resting state[17–19], as well as the changes in mean arterial pressure (MAP) signals[20, 21]. The spontaneous oscillations of the brain can interact mutually with other physiological oscillations, which are called neurophysiological interactions[22]. To date, the relationship between spontaneous cerebral oscillations (i.e., delta [HbO_2_]) and cardiovascular parameters (e.g., MAP) is a promising technique for the noninvasive assessment of CA status. CA is a stochastic, nonlinear dynamic process[23, 24]. The methods based on correlation, phase shift, or transmission have been widely used in assessing CA. Despite their appealing simplicity and clinical significance, these methods are incapable of taking into account the nonlinear effects. Meanwhile, the transfer function analysis treated CA as a stationary, linear process and averaged out all the potential useful time information[25]. Wavelet transform can separate signal components, provide localized phase information, and analyze the nonstationary aspects of the physiological system[26]. Based on wavelet transform, Rowley et al. evaluated the CA by calculating wavelet cross-correlation between the MAP and O2Hb signals[20]. However, such methods provide no information about causality or about the direction of coupling relationship[27]. One way to characterize the networks of interacting oscillators is through reconstructing the coupling function based on dynamical Bayesian inference (DBI), which amounts to much more than just a new way of investigating correlations. Coupling function can specify the physical rules of interactions between the oscillators. It determines the possibility of qualitative transitions between the oscillations and can describe the functional contribution from each separate subsystem within a single coupling relationship (effective coupling interaction)[28]. The coupling function is capable of detecting the effective phase connectivity within networks of time-evolving coupled phase oscillators subjected to noise[27]. Based on wavelet transform, coupling functions can be reconstructed between interacting oscillations from measured data based on a model of coupled phase oscillators and DBI[29–33]. This method is suitable for the treatment of nonstationary time series, which often exist in physiological signals, such as BP and delta [HbO_2_] signals. Previous studies have evaluated the effective coupling interactions based on coupling functions among different physiological indices, such as neuronal, cardiorespiratory, cardiac, respiratory, and vascular regulation[34, 35]. However, the effective coupling interaction between the cerebral oscillations (i.e. delta [HbO_2_]) and cardiovascular parameters (e.g., MAP) have not been demonstrated.

For a comprehensive assessment of CA, the delta [HbO_2_] signals measured by multi-channel NIRS and ABP signals were obtained simultaneously to analyze the autoregulation capacity in different brain areas. The prefrontal cortex (PFC) is important in planning complex cognitive behaviors, personality expression, and decision-making[36]. It also involved in the maintenance of the attention-demanding balance tasks[37, 38]. The parietal lobe (PL) is mainly responsible for coordinating sensory and motor functions[39]. It also plays an important role in motor dysfunction recovery. Meanwhile, the occipital lobe (OL) is crucial in coordinating language, perception, and abstraction functions and essential in processing visual information[40–42].

The present study focuses on the effective coupling relationship between MAP and delta [HbO_2_] in the cerebral cortex to study the effect of stroke on CA in a group of patients suffering from cerebral infarction (CI), as compared with the healthy controls. In this study, the coupling between MAP and cerebral delta [HbO_2_] is not CA but a marker of it. We hypothesized that the effective coupling interactions between the MAP and delta [HbO_2_] signals in various frequency bands will be altered in patients with stroke. In this study, we reconstructed the coupling functions between the oscillators based on a model of coupled phase oscillators and the DBI method. By quantitative assessment of strength of the couplings, we would reveal an evidence of the alterations in CA caused by stroke and the functional mechanism underlying the interactive system. Our results will provide new insight into the regulation mechanisms of the brain and contribute to the development of therapeutic interventions in patients with stroke.

## Methods and materials

### Subjects

A total of 34 elderly subjects were recruited from the local rehabilitation center to participate in this study; 17 subjects were patients with CI (9 with left hemiparesis [L–H group]; 8 with right hemiparesis [R–H group]), and 17 healthy controls [healthy group]). Patients with clinical and CT diagnosis of first-ever stroke were included in this study. All measured subjects had systolic BP less than 150 mmHg and diastolic BP less than 90 mmHg and body mass indices less than 30 during the recording. Healthy subjects showed no history of any neurological or vascular disease. Healthy subjects were excluded from the study if they were diagnosed with hypertension, diabetes mellitus, subarachnoid hemorrhage, insufficiency of heart, lung, kidneys, or liver function, as well as smoking or drinking habits, and if they are taking additional medications. Diabetes mellitus was diagnosed based on the clinical assessment or fasting serum glucose level. Table 1 shows the basic information of participants, including age, weight, height, BP, and MMSE scores. Table 2 shows the detailed clinical characteristic of patients with stroke. Written informed consent was obtained from the participants. The experimental procedure was approved by the Human Ethics Committee of National Research Center for Rehabilitation Technical Aids and was in accordance with the ethical standards specified by the Helsinki Declaration of 1975 (revised in 2008).

**Table 1:** Characteristics of the participants.

Characteristic	Healthy Controls	Cerebral Infarction		*p* for difference		
		R–H	L–H	*p*^1^	*p*^2^	*p*^3^
Age (years)	51.8(7.9)	53.2(12.6)	57.2(9.1)	0.774	0.663	0.580
BMI	24.2(4.5)	22.6(2.8)	23.4(1.7)	0.370	0.600	0.504
Female sex	47%	25%	11%	0.294	0.067	0.453
Systolic blood pressure	130.8(11.2)	127.9(25.9)	140.3(15.8)	0.761	0.089	0.263
Diastolic blood pressure	75.9(11)	74.5(11.1)	78.7(8.9)	0.727	0.529	0.376
MMSE	26.8(2.3)	24.3(5.8)	24.9(4.6)	0.358	0.282	0.840

Values are presented as means and standard deviations (SD) and percentages. *p* values are calculated with t-test for means and SD and Chi-square test for percentage. MMSE: Mini-Mental State Examination. *p*^1^ for the difference between healthy group and R–H group. *p*^2^ for the difference between healthy group and L–H group. *p*^3^ for the difference between R–H group and L–H group.

**Table 2:** Clinical characteristic of patients with stroke.

Patient NO.	Sex	Age, Years	Onset Time, Month	Hemiplegia	Location of Lesion	Hypertension	Diabetes mellitus
1	M	49	5	L	R basal ganglia; R oval	Y	Y
2	M	61	3	L	R pons; R basal ganglion	Y	Y
3	M	62	5	L	R basal ganglion; Thalamus	Y	N
4	F	74	4	L	R pons; R parietal lobe	Y	N
5	M	61	3	L	R pons; R basal ganglion	Y	N
6	M	49	5	L	R Frontal, R Parietal, R Temporal lobe	Y	N
7	F	62	5	L	R Frontal, R Parietal, R Temporal lobe; R temporal-occipital junction	N	Y
8	M	45	6	L	R Frontal, R parietal, R temporal lobe	Y	N
9	M	52	5	L	R oval; R basal ganglia	Y	N
10	F	30	5	R	L basal ganglia	Y	Y
11	M	57	2	R	L parietal lobe	Y	N
12	M	67	3	R	L basal ganglia	N	N
13	M	46	3	R	L basal ganglia; L frontal lobe; L insula	N	Y
14	M	46	5	R	L Frontal, parietal, temporal lobe	Y	Y
15	F	69	5	R	L thalamus; Bilateral basal ganglia; L frontal lobe	Y	Y
16	M	54	6	R	L frontal lobe, L temporal lobe, L parietal lobe, insular; basal ganglia	Y	Y
17	M	57	3	R	L basal ganglia; L insula; L frontal lobe	N	Y

This table shows clinical characteristic of patients with stroke. F, female; M, male; R, right; L, left; Y, yes; N, no

### Measurement

**Fig 1:**
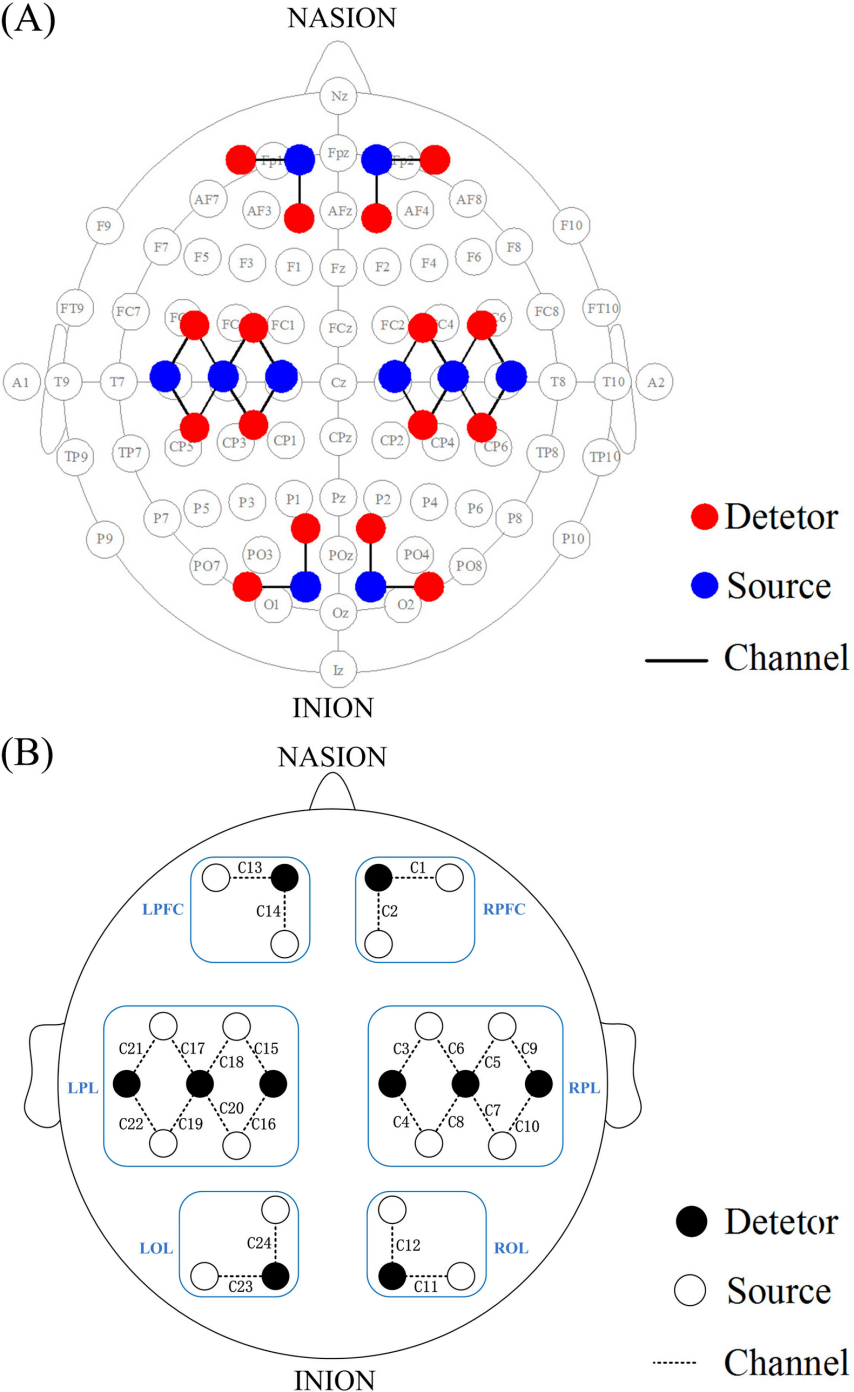
Configuration of source optodes, detector optodes and channels. (A) 24 channels corresponding to the 10/10 system. (B) Six cerebral cortex areas are separated by the rectangle frame as LPFC, RPFC, LPL, RPL, LOL and ROL. The ‘C’ represents channel.

Simultaneous measurements of delta [HbO_2_] and ABP signals were recorded for 10 min in the resting state. After the basic information of the subjects was recorded, the measurements were performed on the subjects in their comfortable supine position to minimize head and wrist movements. NIRS measurements were performed using a 24-channel cerebral tissue saturation monitor (NirScan Danyang Huichuang Medical Equipment CO. Ltd) with three wavelengths: 755, 808, and 855 nm. The distance between the detector and the source was 30 mm. Delta [HbO_2_] was calculated using the modified Beer–Lambert law[43]. The sampling rate was 10 Hz. A total of 24 channels were positioned on the left PFC (LPFC), right PFC (RPFC), left PL (LPL), right PL (RPL), left OL (LOL) and right OL (ROL) in accordance with the International 10/10 System (Fig 1). Using the calibration function of the instrument and the corresponding template, each channel arrangement can accurately correspond to the 10/10 electrode positions according to different head sizes. The placement of the monitors was concretely described previously[44]. Before the measurements, the sensors were secured with a black elastic plaster wrapped around the forehead to ensure no intrusion of background light[45].

Continuous ABP signal was monitored with a noninvasive pressure sensor attached to the wrist to obtain the ABP signal using an analysis system (FDP-1, Shanghai Science Teaching Co., China) at a sampling frequency of 1 000 Hz. This system continuously measured the ABP signals by using a sensor located on the subject’s radial artery.

dx.doi.org/10.17504/protocols.io.ntkdekw

### Data preprocessing

Systolic and diastolic BP were extracted from the ABP signal by applying a gradient function, which can automatically detect the signal peaks and troughs of the signals[46]. This algorithm involves an automatic and adaptive rule-based search for the large gradient selected as a reference peak in BP due to the systole, followed by the waveform integration on the heartbeat cycle. The MAP was calculated as the diastolic pressure plus 1/3 of the pulse pressure. MAP was resampled to 10 Hz using cubic spline interpolation to achieve a uniform time base.

**Fig 2:**
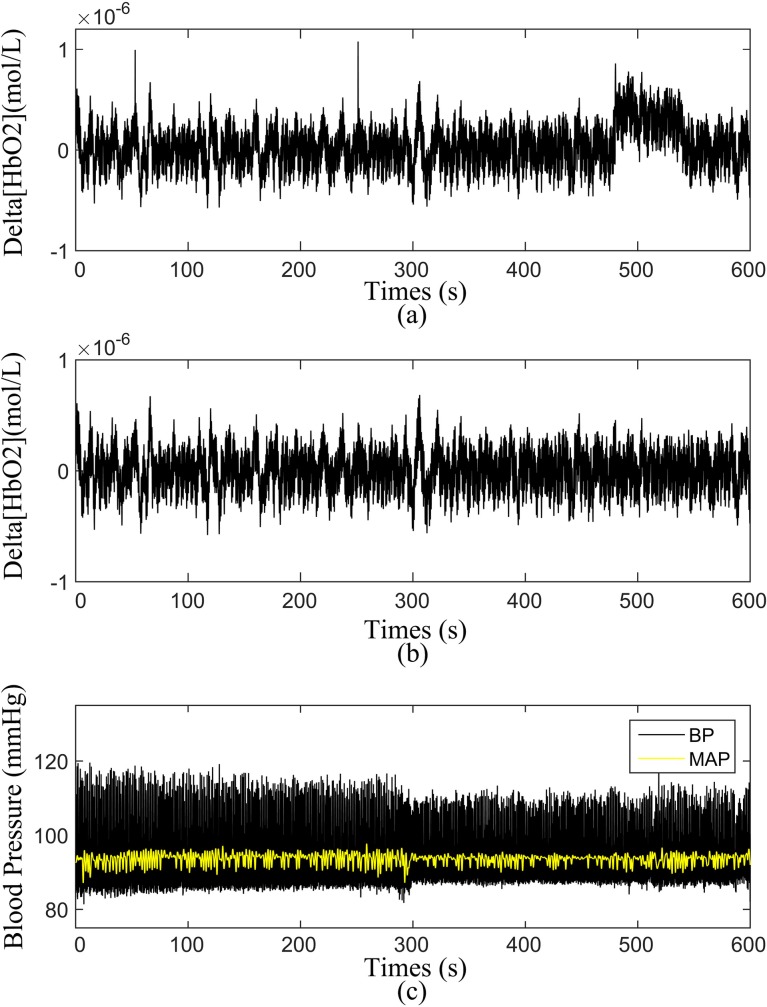
Time series of the simultaneous recordings of ABP signals and Delta[HbO2] signal from one subject. Delta [HbO2] time series before **(a)** and after **(b)** pre-processing. **(c)** measured BP and MAP time series.

First, the moving average method was applied to remove the noise-like abrupt spikes caused by the movement artifacts or background light, involving an algorithm based on moving standard and spine interpolation routines[47, 48]. After the removal of abnormal points, the delta [HbO_2_] and MAP signals were band-pass filtered with a Butterworth filter at a low cut-off frequency of 0.005 Hz to remove extremely slow variations and a high cutoff frequency of 2 Hz to remove the uncorrelated noise components. The upper limit of 2 Hz was set to include the heart rate frequency, whereas the lower limit was selected to include the possible regulatory mechanisms of the tissue oxygenation signal[45, 49]. The method used for data preprocessing has been described in detail in our previous studies[50]. Prior to the wavelet analysis, the delta [HbO_2_] and MAP signals were normalized to avoid the systematic differences between the subjects and groups. Fig 2 shows the typical time series of the measured delta [HbO_2_], BP, and MAP.

### Wavelet transforms

Wavelet transform is a method that provides the complex transformation of a time series from the time to time-frequency domain, containing both the phase and amplitude dynamics of the oscillatory components in the signals[51].

$$W(s,t)=\frac{1}{\sqrt{s}}\cdot \int_{-\infty }^{\infty }\psi (\frac{u-t}{s})g(u)du$$ (1)

The mother wavelet *Ψ* in this paper is the Morlet wavelet. *s* is the scaling factor and its corresponding frequency is *f* = 1/*s*. With the complex Morlet wavelet, the wavelet coefficients *w*_*k*_(*t*_*n*_) are complex numbers in the time-frequency domain[52].

$$\omega_{k}(t_{n})=W_{k}(f,t_{n})∙e^{i\phi_{k}(f,t_{n})}=a_{k}(f,t_{n})+ib_{k}(f,t_{n})$$ (2)

The equation above defines the instantaneous relative phase *ϕ*_*k*_(*f*,*t*_*n*_) for each frequency (*f*) and time (*t*_*n*_)

$$\phi_{k}\left(f,t_{n}\right)=\arctan\left[\frac{b_{k}\left(f,t_{n}\right)}{a_{k}\left(f,t_{n}\right)}\right]$$ (3)

Phase dynamic information can be used to investigate the effective coupling relationships among the oscillations from different signals in difference time scales[53]. Spontaneous oscillations of NIRS and MAP signals in various characteristic frequency bands with logarithmic frequency resolution have been identified by wavelet analysis. The coupling functions between the delta [HbO_2_] and MAP oscillators were analyzed in four frequency intervals (I, 0.6Hz to 2 Hz; II, 0.145Hz to 0.6 Hz; III, 0.052 Hz to 0.145 Hz; IV, 0.021 Hz to 0.052 Hz).

### Inference of frequency-specific coupling function

Coupling functions can describe the causality mechanism of interactions between a pair of oscillators[27]. DBI can infer time-evolving coupled dynamics in the presence of noise[29]. The signals derived from the specific-frequency intervals are oscillations, and their interactions can be investigated by focusing on their phase dynamics[54, 55]. To determine the coupling relationship from measured signals, the system is modeled as a network of *N* coupled phase oscillators. The system of *N* stochastic differential equations with time-varying parameters is defined as[27]:

$$\dot{\emptyset_{i}}(t)=\omega_{i}(t)+q_{i}(\emptyset_{i},\emptyset_{j,}\emptyset_{k},\ldots ,\emptyset_{N},t)+\xi_{i}(t)$$ (4)

where *i* = 1,…,*N*, *$\dot{\emptyset_{i}}$* is the instantaneous frequency of each oscillator, which is determined by the combination of its natural frequency (*ω*_*i*_) and a function (*q*_*i*_) of all the *N* oscillators’ phases (*∅*_1_,…,*N*) representing the coupling configuration. In the stochastic part of Eq (4), *ξ*_*i*_ is the Gaussian white noise. The deterministic periodic part of Eq (4) can be Fourier-decomposed for each oscillator into a sum of base functions, Φ_*k*_ = esp[*l*(*k*_1_*ϕ*_1_ + *k*_2_*ϕ*_2_ + ⋯ + *k*_*N*_*ϕ*_*N*_)], characterized by a set of time-varying parameters $\left(c_{k}^{\left(i\right)}\right)$:

$$\dot{\emptyset_{i}}(t)=\sum_{k=-K}^{K}c_{k}^{\left(i\right)}\Phi_{k}(\phi_{1},\phi_{2},\ldots ,\phi_{n})+\xi_{i}(t)$$ (5)

where *K* is the order of the Fourier expansion. In this study, set *K* = 2. Eq (5) as the phase dynamics extracted from the time series χ = {*x*_*l*_ ≡ *x*(*t*_*l*_)} (*t*_*l*_ = *lh*), with *l* = 1,…,*L*, as the set of parameters characterizing the network $M=\{c_{k}^{\left(i\right)},D_{r,s}\}$, which can completely describe the couplings ($c_{k}^{\left(i\right)}$) and noise (*D*_*r*,*s*_) of the network, is to be inferred by DBI. Bayesian approach enables us to evaluate the posterior density, $p_{\chi }(M|X)$, of the unknown matrix of parameters M from time series (X), given a prior density, $p_{prior}(M)$, by building a likelihood function l(X|M), as shown in Eq (6):

$$p_{\chi }(M|X)=\frac{l(X|M)p_{prior}(M)}{\int l(X|M)p_{prior}(M)dM}$$ (6)

Given the time series X are provided, and the Fourier components (Φ_*k*_) act as base functions. The main task for DBI is to infer the unknown model parameters $\left(c_{k}^{\left(i\right)}\right)$ through which the coupling relationship are evaluated[56]. The likelihood function is computed through the stochastic integral of noise term over time; the negative log-likelihood function S=−lnl(X|M), is given as:

$$\begin{array}{l} S=\frac{L}{2}\ln\left|D\right|+\\ \frac{h}{2}\sum_{l=0}^{L-1}\left(c_{k}\frac{\partial \Phi_{k}\left(\phi .,l\right)}{\partial \phi }+{\left[\dot{\phi_{l}}-c_{k}\Phi_{k}\left(\phi_{.,l}^{*}\right)\right]}^{T}\left(D^{-1}\right)\left[\dot{\phi_{l}}-c_{k}\Phi_{k}\left(\phi_{.,l}^{*}\right)\right]\right) \end{array}$$ (7)

where $\dot{\phi_{l}}=(\phi_{l+1}-\phi_{l})/h$. The dot index in *ϕ*_.,*l*_ represents the relevant index. If the prior probability of parameters M is a multivariate normal distribution, and the likelihood (8) is of quadratic form, so will be the posterior probability. In this particular distribution for the parameter c, with mean $\bar{c,}$ and covariance matrix ∑_*prior*_, the final stationary point of *S* is calculated recursively based on a tutorial on time-evolving DBI and software codes from the following equations[56]:

$$\begin{array}{c} D=\frac{h}{L}{\left(\dot{\phi_{l}}-c_{k}\Phi_{k}\left(\phi_{.,l}^{*}\right)\right)}^{T}\left(\dot{\phi_{l}}-c_{k}\Phi_{k}\left(\phi_{.,l}^{*}\right)\right),\\ \Gamma_{W}={\left(\Xi_{prior}\right)}_{kw}+h\Phi_{k}\left(\phi_{.,l}^{*}\right)\left(D^{-1}\right)\dot{\phi_{l}}-\frac{h}{2}\frac{\partial \Phi_{k}\left(\phi .,l\right)}{\partial \phi },\\ \Xi_{kw}={\left(\Xi_{prior}\right)}_{kw}+h\Phi_{k}\left(\phi_{.,l}^{*}\right)\left(D^{-1}\right)\Phi_{w}\left(\phi_{.,l}^{*}\right),\\ c_{k}={\left(\Xi^{-1}\right)}_{kw}\Gamma_{W}, \end{array}$$ (8)

where summation over *l* = 1,…,*L*, and over the repeated indices *k* and *w*, is implicit. Once we have the inferred parameters *c*, we can calculate the coupling quantities and characteristics. The coupling functions are evaluated on a 2π × 2π grid using the Fourier components (Φ_*k*_) as relevant base functions.

To facilitate comparisons between coupling functions, coupling strength (CS), *σ*_*i*,*j*_, defined as the Euclidean norm of the inferred parameters from the phase dynamics is calculated as:

$$\sigma_{i,j}=\sqrt{\sum_{k=-K}^{K}{\left(c_{k}^{\left(i:j\right)}\right)}^{2}},$$ (9)

The CS quantifies the coupling amplitude. It corresponds to the coupling from the oscillators *σ* to the oscillator *i*. In this paper, by quantitative assessment of the forms and strength of couplings, we can reveal the alterations in CA caused by stroke.

### Surrogate analyses

To study whether the obtained CS is significant, we used surrogates to validate the results for the coupling functions[57]. A total of 100 independent surrogates were generated by randomizing the phase signal from each signal but preserving the statistical characteristics of the original networks[35]. We did not consider the relationship if it did not exhibit a significant difference compared with the random result created by the surrogates. Coupling from delta [HbO_2_] to MAP direction was weaker and usually insignificant compared with the result calculated by surrogates. Thus, we only presented the coupling functions in the predominant direction from MAP to delta [HbO_2_] in four frequency intervals.

A total of 24 effective coupling interactions in each frequency interval were detected from MAP to delta [HbO_2_] in 24 channels in each subject. The CS from MAP to delta [HbO_2_] represents the influence that the changes in BP exerted on the delta [HbO_2_]. The high CS may be an evidence of the impaired CA in the brain, whereas low CS indicates the intact autoregulation.

### Statistical analysis

The characteristic values of age, body mass index, BP, and sex were expressed as the mean (SD) or percentages. Kolmogorov Smirnov and Levene tests were applied to test the variance normality and homogeneity of the data at the group level. Significant differences of the characteristic values were determined using *t*-test for means and SDs, and chi-square test for percentages. The one-way ANOVA was performed on the channel-wise CS between the patients with stroke and healthy controls. Student’s paired t tests were used in comparing the differences within affected and non-affected hemispheres in L–H and R–H groups. The Bonferroni correction was applied to the *p*-values for the multiple comparisons. Totally there were 4 × 2 = 8 inter-groups pair-wise comparisons (healthy group and L–H group, healthy group VS R–H group, four frequency intervals), thereby the corrected p-value threshold was set at *p* < 0.006.

## Results

### Demographic data

Participant characteristics are shown in **Table 1**. Sex and age were matched between the groups. No significant difference was observed in the demographic data between the three groups.

### Effect of stroke on the coupling function

#### Channel-wise CS from MAP to delta [HbO_2_]

**Fig 3:**
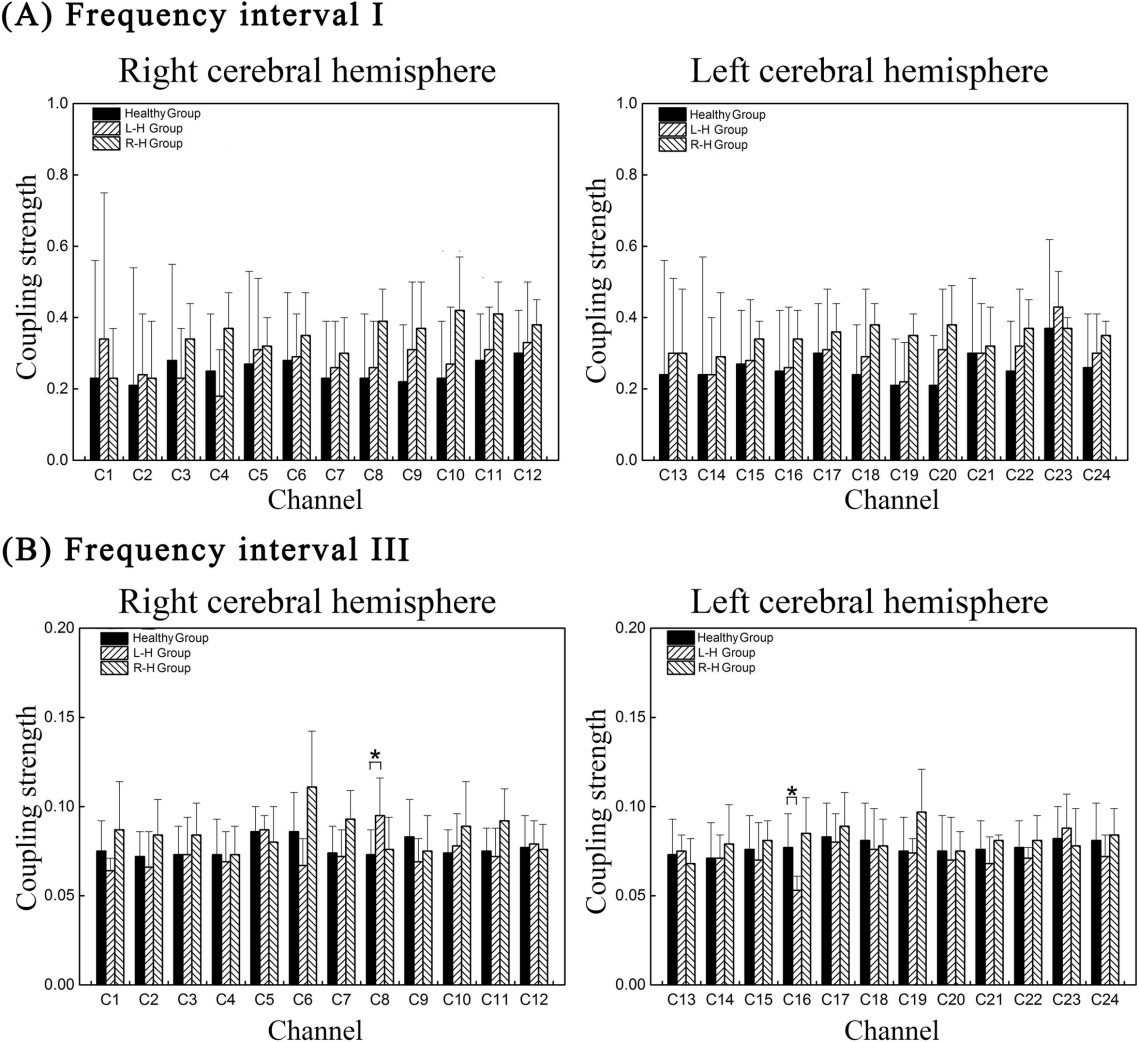
Group statistical analysis in channel-wise CS from MAP to delta [HbO2] in different frequency interval. (A) channel-wise CS in frequency interval I (B) channel-wise CS in frequency interval III. Each column shows the CS mean and SD of a specific effective coupling interaction for different groups. The healthy, L–H and R–H groups are represented by different columns as illustrated by the legend in the figure. The line connectors and ‘*’ on the top of individual columns indicate that the difference between the two column distributions was statistically significant.

Fig 3 shows the comparison of channel-wise CS of 24 effective coupling interactions in four frequency intervals among the groups. In interval III, compared with healthy group, the CS from MAP to delta [HbO_2_] in C8 (*p* = 0.003) significantly increased in L–H group. However, significant decrease in CS from MAP to delta [HbO_2_] was found in C16 (*p* = 0.001) in L–H group in interval III.

#### Region-wise coupling function from MAP to delta [HbO2]

**Fig 4:**
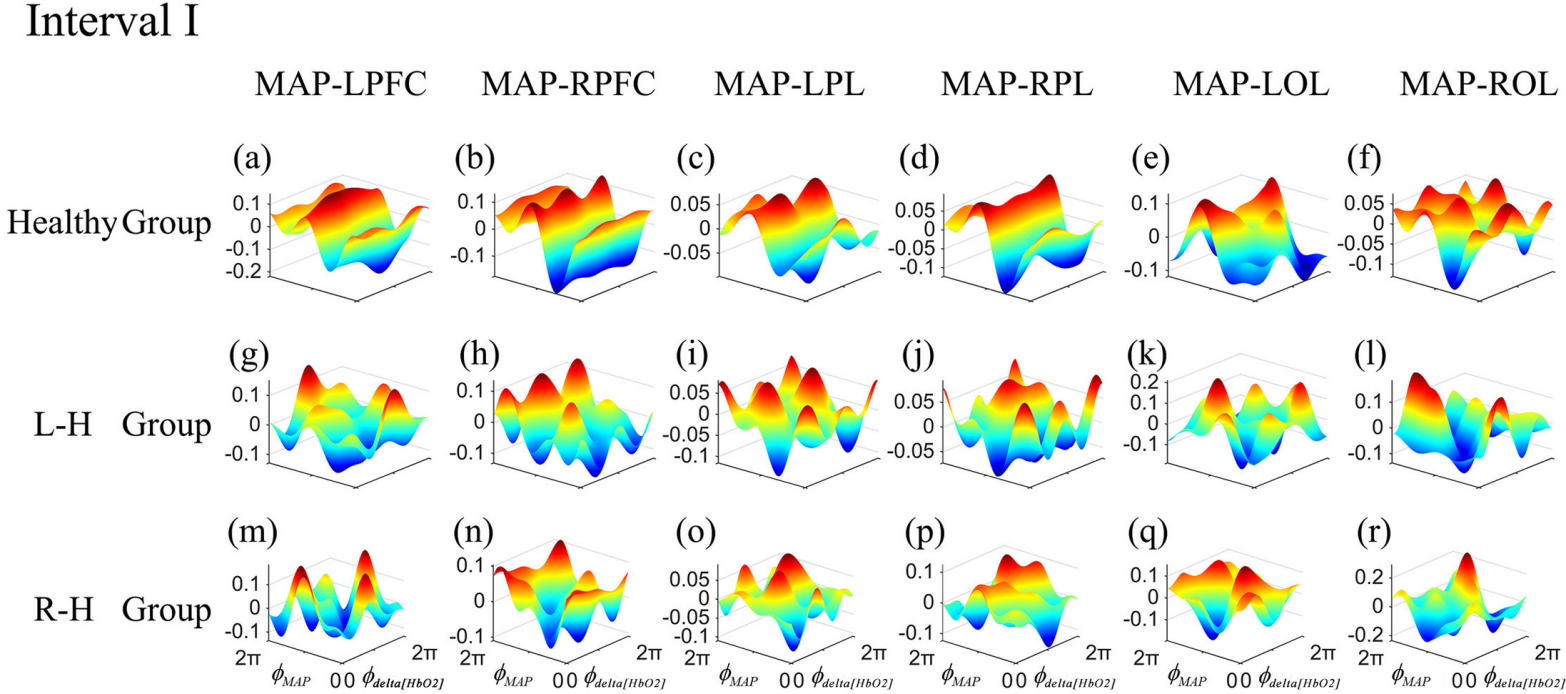
Group-average coupling functions *q*_*delta*[*HbO2*]_(*∅*_*delta*[*HbO2*]_,*∅*_*MAP*_) between MAP and delta [HbO_2_] oscillations in LPFC, RPFC, LPL, RPL, LOL and ROL in interval I. The coupling functions describe the functional influence from the MAP to delta [HbO_2_] oscillator in different brain regions.

**Fig 5:**
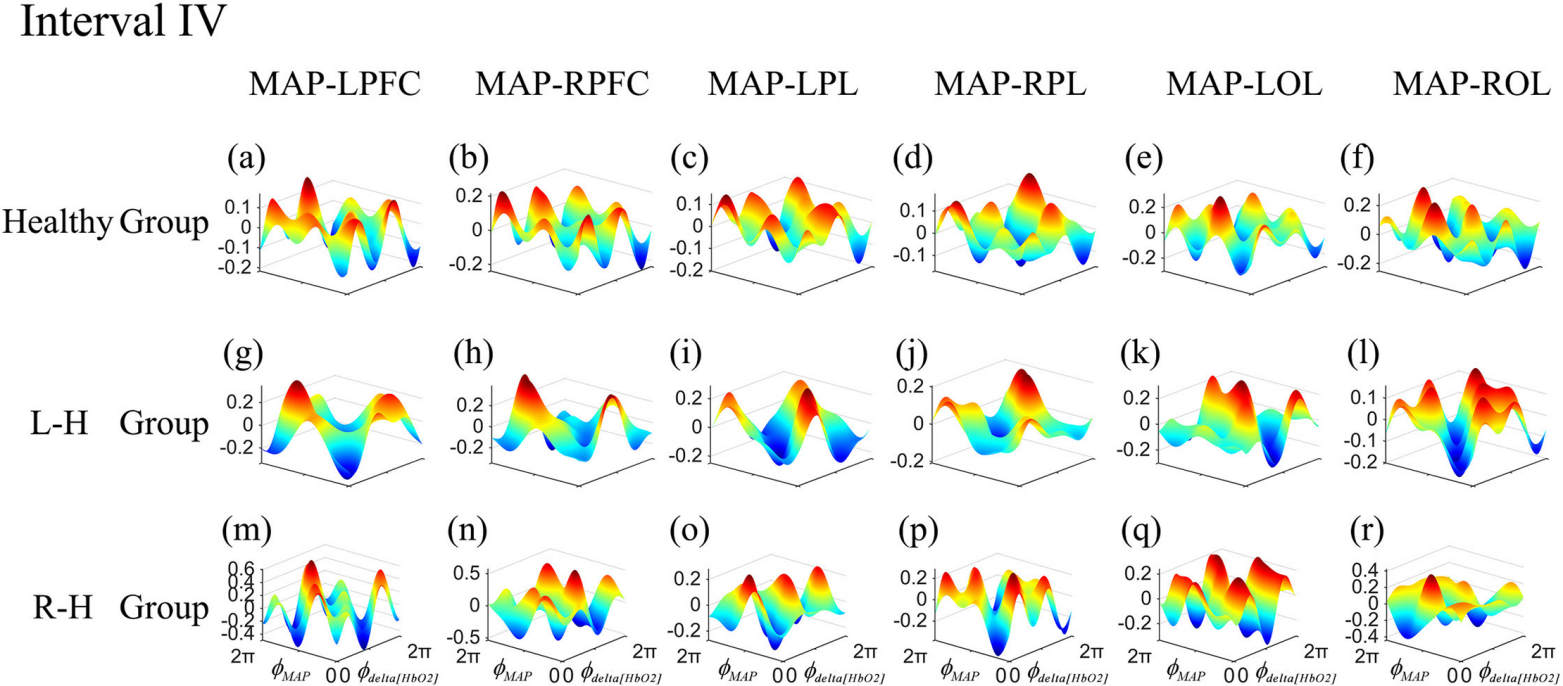
Group-average coupling functions *q*_*delta*[*HbO2*]_(*∅*_*delta*[*HbO2*]_,*∅*_*MAP*_) between MAP and delta [HbO_2_] oscillations in LPFC, RPFC, LPL, RPL, LOL and ROL in interval IV. The coupling functions describe the functional influence from the MAP to delta [HbO_2_] oscillator in different brain regions.

The frequency-specific coupling functions for every measured brain region were obtained by using averaging method. According to the distribution of 24 channels in 6 brain regions, six coupling functions of each subject were obtained by averaging the coupling functions of the internal channels in each brain cortex. In one frequency interval, the coupling functions of 24 directed coupling pairs were averaged over 6 directed coupling interaction types: MAP→LPFC, MAP→RPFC, MAP→LPL, MAP→RPL, MAP→LOL, and MAP→ROL. To gain further insights into the coupling nature from MAP to delta [HbO_2_] in different frequency intervals, we analyzed the form of the reconstructed coupling functions, *q*_*delta*[*HbO2*]_(*∅*_*delta*[*HbO2*]_, *∅*_*MAP*_), of all effective coupling interactions in each group. The coupling function can describe the influence of oscillator *∅*_*MAP*_ on the oscillator *∅*_*delta*[*HbO2*]_. In this paper, we only exhibited the group-average coupling functions of the frequency interval with differences between groups. Figs 4 and 5 show the group-average forms of coupling in intervals I and IV for the healthy group ((Fig 4A–4F) and (Fig 5A–5F)), L–H ((Fig 4G–4L) and (Fig 5G–5L)), and R–H ((Fig 4M–4R) and (Fig 5M–5R)). The form of the functions in healthy group in interval I ((Fig 4A–4D)) appears as a sine wave, which changes predominantly along the *∅*_*MAP*_ axis. When the MAP oscillator was between π and 2π, the coupling functions were higher which means the MAP activity accelerated the delta [HbO_2_] oscillations. The coupling functions in L–H group and R–H group showed in complex and varying form, which changes along both axes. The coupling functions in L–H group ((Fig 4K–4L)) and R–H group ((Fig 5O–5R)) showed higher amplitude than healthy group. The coupling functions of each group in interval IV showed a complicated and varying form. The coupling amplitudes in some interactions increased in the L–H group ((Fig 5G–5J)) and R–H group ((Fig 5M–5R)), compared with healthy group. The R–H group had the largest coupling amplitude in the coupling functions, implying that the *q*_*delta*[*HbO2*]_(*∅*_*delta*[*HbO2*]_, *∅*_*MAP*_) coupling depended more on the MAP oscillator.

#### Difference in CA between affected and unaffected sides

The frequency-specific CS for every measured brain region was obtained by using averaging method. Based on the statistical test, no significant difference between affected and unaffected sides was observed in L-H and R-H groups (S1 Table). These results indicated there was a global impairment of CA in patients with stroke.

## Discussion

In response to a change in the perfusion pressure, the active autonomic neural control of the cerebral circulation and an adaptation in cerebrovascular resistance cause CBF to return to its baseline[3, 58, 59]. This process can be analyzed through the effective coupling interactions between delta [HbO_2_] in the cortical and MAP oscillations on a wide range of time scales. In this study, we analyzed the frequency-specific effective coupling interactions between delta [HbO_2_] measured by NIRS and MAP based on the coupling functions and DBI for patients with stroke and healthy controls. In L–H group, the CS from MAP to delta [HbO_2_] in interval III in C8 was significantly higher than that in healthy group, whereas the CS from MAP to delta [HbO_2_] in C16 was significantly lower than that in healthy controls. Based on the form of group-average coupling functions in different brain regions, the coupling in MAP→delta [HbO_2_] showed higher amplitude in interval I and IV in patients with stroke than that in healthy controls. The increased CS and coupling amplitude may be an evidence of impairment in CA in patients with stroke. No significant difference in CS between affected and unaffected sides was observed in the patients with stroke, indicating a global impairment of CA. These results provide new insight into the regulation mechanisms and contribute to the development of therapeutic interventions in patients with stroke.

The oscillations in intervals I and II, corresponding to the cardiac and respiratory activities, served as pumps to provide blood through the vessels, which has been manifested in the vessels[60]. The cardiac and respiratory activities belong to the high-frequency oscillations, which are beyond the control of CA[3]. The regulation of CBF is an integrative process that involves the marked influence of cardiovascular function[61]. The regulation of cerebral delta [HbO_2_] required time to initiate the cerebrovascular interactions to adapt to the changes in the perfusion pressure. Compared with healthy group, the coupling in MAP→delta [HbO_2_] showed higher amplitude in patients with stroke. These results indicated a stronger influence that MAP oscillations exerted on delta [HbO_2_] in the brain in patients with stroke than in healthy controls. Higher coupling amplitude from MAP to the brain indicated that the transmission of BP changes in cardiac activity to CBF was faster in patients with stroke than in healthy controls. Thus, shorter time delay was observed in the R–H group to initiate the cerebrovascular adaptations, thereby resulting in the insufficiency of the brain in patients with stroke to cope dynamically with rapid BP changes. These results were consistent with the assumption that even a minor fall in perfusion pressure may result in a fall in CBF[62]. CA may impact functional recovery following stroke and the better CA function is associated with better functional status. The channels with increased coupling amplitude are mainly distributed in RPL and ROL, respectively. The increased effects that changes of MAP on the delta [HbO_2_] might affect the brain function of these brain regions, leading to motor dysfunction and the decreased coordination between these brain regions.

The oscillation analyzed in vascular dynamics in frequency III was suggested to originate locally from the intrinsic myogenic activity of smooth muscle cells in the resistance vessels[63]. This myogenic mechanism may be partly under autonomic control associated with the changes in the peripheral sympathetic nerve activity[60, 64]. This mechanism is related to spontaneous movements of smooth muscle in the vessel wall, which is also called vasomotion[65]. The vascular smooth muscles contract or relax, involving the opening and closing of ion channels in the endothelial and smooth muscle cells in the vessel walls in response to the change in intravascular pressure[66, 67]. The spontaneous low-frequency oscillations in cerebral hemodynamics may lie in the regulation of regional CBF change and the intrinsic myogenic activity of smooth muscle cells in interval III acts to buffer small changes in CBF[68]. The vascular resistance were altered in low frequency (LF) arteriole vessel, which affects the net pressure drop across the vessels[69]. The increased vessel stiffness is an independent risk factor to the development of stroke[70]. In L–H group, the results showed that the CS from MAP to delta [HbO_2_] in C8, which was distributed in the affected side, was significantly higher than that in healthy group. The significantly increased CS from MAP to delta [HbO_2_] in interval III might indicate increased vessel stiffness of the smooth muscle cells, indicating an evidence of the impaired CA in patients with stroke. The increased vessel stiffness in the stroke indicated a reduced contractility and vasodilation capability of the smooth muscle cells and failed to adjust the blood flow to satisfy the oxygen requirement of cells. Previous study based on NIRS has demonstrated that patients with multiple infarctions showed a significantly reduced vasomotor reactivity in the cerebral cortex and the reduction of vasomotor reactivity is thought to play an important role in the pathogenesis of cerebral microangiopathy[71]. However, compared with the healthy controls, the L–H group showed significantly decreased CS in C16 which is distributed in the unaffected side. This result may suggest that the regulation capacity of myogenic activity was enhanced in unaffected hemisphere. This result might indicate that there was compensatory function in the regulation of regional CBF in patients with stroke.

Within the brain, hemodynamic parameters are closely regulated through the tight neurovascular coupling and partial autonomic control in the frequency interval IV[2]. The nervous system maintains the basal level of contraction of the vessels. As previously mentioned, changes in the steady-state cerebrovascular resistance or vascular compliance or both may influence the beat-to-beat changes in CBF during the steady-state CA[72, 73]. During the orthostatic stress, the presence of cerebral vasoconstriction is associated with the augmented sympathetic nerve activity[74]. The sympathetic pathways from the brain to the cerebral blood vessels must be intact for normal CA to occur[62]. It has been proved that the neurogenic activity of the cerebral circulation regulates the beat-to-beat CBF in humans[2]. In interval IV, no significant difference was observed in CS from MAP to delta [HbO_2_] between patients with stroke and healthy controls. The result indicated that the capacity of neurogenic activity to regulate the beat-to-beat CBF reserved in patients with stroke.

In this study, the significantly increased CS from MAP to delta [HbO_2_] in interval III indicated an impaired CA in patients with stroke. These results were consistent with the conclusion that normal cardiovascular homeostatic mechanisms are impaired in ischaemic stroke[70]. As a result, CBF tends to depend directly on systemic BP. The increase in BP may render the brain at risk for cerebral oedema or hemorrhagic transformation of the infarct, whereas reducing systemic BP can reduce flow to the ischemic penumbra and increase infarct size.

In the patients with stroke, the lack of a significant difference in region-wise CS from MAP to delta [HbO_2_] between the affected and unaffected sides indicated a global impairment of CA with stroke (S1 Table). Table 2 showed that the patients with stroke participated in this study had multiple subcortical infarcts. The impaired autoregulation could be demonstrated in the nonaffected hemisphere[7, 75] Schwarz et al. found significant changes in the unaffected hemisphere, suggesting bilaterally impaired autoregulation in severe stroke[76, 77].These results were consistent with previous studies that the impairment in CA is not confined to the affected hemisphere[76, 77].

### Method consideration

In general, Bonferroni correction was used for multiple comparisons. The Bonferroni correction controls the experiment-wise alpha well and has strong control of Type I error. A basic assumption for choosing the Bonferroni correction method for multiple comparisons is that the test variable is independent[78]. The NIRS-based neuroimaging technique measures the cortical blood flow and mainly reflects the microvasculature activity of brain[79]. There would be a highly correlation between fNIRS signals among the channels because they contain global vascular response arising from the blood flow and oxidative metabolism[80, 81]. So, the CS of 24 channel-wise derived from NIRS-based signals in the brain was not independent. Moreover, for a large of comparisons, the Bonferroni correction becomes very conservative and results in a large number of type II errors and greatly diminished power to detect differentiation among pairs of sample collections. In this study, one-way ANOVA was performed on the channel-wise CS between the patients with stroke and healthy controls. In each frequency interval, two groups for CS comparison were designed (Group healthy VS Group L-H, Group healthy VS Group R-H). So totally there are 4 × 2 = 8 inter-groups pair-wise comparisons. Therefore, Bonferroni correction was applied to these multiple comparisons and the corrected p-value threshold was set at p < 0.006.

### Limitation

In this study, we measured the delta [HbO_2_] by NIRS. NIRS provides a surrogate of fluctuations in CBF. NIRS signals obtained at several cortical regions mainly reflect the spontaneous regional hemodynamic fluctuations that originate from spontaneous cortical activity during the resting state[82]. However, one primary challenge in measurement is the high sensitivity to hemodynamic fluctuations of NIRS. NIRS photons must penetrate the superficial tissue layers (scalp and skull) before reaching the cortex to monitor the evoked brain activity. Therefore, a limitation of the present study is that the measured signals will inevitably be contaminated by the noise as well as nonspecific hemodynamic variations provided by the scalp and skull[83–85]. In this paper, we used the continuous wavelet transform to divide the signals into four frequency intervals to study the frequency-specific coupling interactions between the brain and BP. In further studies, short source-detector separation channels will be used to filter the interference of scalp and skull signals.

The group of patients with stroke that participated in this work was classified according to the left and right hemiplegia. Severe extracranial or intracranial artery stenosis and clinical conditions (e.g., chronic hypertension, diabetes mellitus, and silent infarcts) may confound the assessment of CA. Some clinical conditions may disrupt myogenic mechanisms, whereas others may impair autoregulation by blocking the metabolic or neurogenic pathways[58]. Additional knowledge is needed about the physiological and pathological determinants of CA in stroke, so that determinants of autoregulation can be better controlled in future studies. These drawbacks deserve further study, which may identify possibilities for therapeutic intervention.

### Conclusion

In this study, the frequency-specific effective coupling interactions between the delta [HbO_2_] and MAP were calculated based on the coupling functions and DBI to assess the CA in stroke. The CS can specify the effects of MAP changes on delta [HbO_2_] to study the alterations of CA mechanism in the patients with stroke suffering from CI. In L–H group, the CS from MAP to delta [HbO_2_] in interval III in C8 was significantly higher than that in healthy group. Based on the form of group-average coupling functions in different brain regions, the coupling in MAP→delta [HbO_2_] showed higher amplitude in interval I and IV in patients with stroke than that in healthy controls. The increased CS and coupling amplitude suggested an impaired CA in patients with stroke. Compared with healthy controls, significant decreased CS from MAP to delta [HbO_2_] in channel 16 (*p* = 0.001) was found in L–H group, which might indicate the compensatory mechanism in CA of the unaffected sides. No significant difference in CS between affected and unaffected sides was observed in patients with stroke, which indicated a global impaired of CA. These findings are important to identify the frequency-dependent properties of dynamic CA and the underlying CA mechanisms in patients with history of stroke. This study provides a method for the assessment of CA and will contribute to the development of therapeutic interventions in patients with stroke.

## Supporting information

S1 Table Differences in CS in patients with stroke between affected hemispheres and unaffected hemispheres. (PDF)

S1 File This is channel-wise CS data of four frequency intervals of healthy group, L–H group and R–H group. (MAT)

S2 File This is coupling function data of four frequency intervals of healthy group, L–H group and R–H group. (MAT)

S3 File This is region-wise CS data of four frequency intervals of healthy group, L–H group and R–H group. (XLSX)

## References

[1] Ronney BP. Assessment of cerebral pressure autoregulation in humans—a review of measurement methods. Physiological Measurement. 1998;19(3):305 9735883 10.1088/0967-3334/19/3/001

[2] Zhang R, Zuckerman JH, Iwasaki K, Wilson TE, Crandall CG, Levine BD. Autonomic neural control of dynamic cerebral autoregulation in humans. Circulation. 2002;106(14):1814–20. 12356635 10.1161/01.cir.0000031798.07790.fe

[3] van Beek AH, Claassen JA, Rikkert MG, Jansen RW. Cerebral autoregulation: an overview of current concepts and methodology with special focus on the elderly. Journal of Cerebral Blood Flow & Metabolism Official Journal of the International Society of Cerebral Blood Flow & Metabolism. 2008;28(6):1071. 10.1038/jcbfm.2008.13 18349877

[4] Katsogridakis E, Bush G, Fan L, Birch AA, Simpson DM, Allen R, et al Detection of impaired cerebral autoregulation improves by increasing arterial blood pressure variability. Journal of Cerebral Blood Flow & Metabolism. 2013;33(4):519. 23232946 10.1038/jcbfm.2012.191 PMC3618385

[5] Tarumi T, Dunsky DI, Khan MA, Liu J, Hill C, Armstrong K, et al Dynamic cerebral autoregulation and tissue oxygenation in amnestic mild cognitive impairment. Journal of Alzheimers Disease Jad. 2014;41(3):765 doi: 10.3233/JAD-132018 24670396 10.3233/JAD-132018

[6] Aries MJ, Elting JW, De KJ, Kremer BP, Vroomen PC. Cerebral autoregulation in stroke: a review of transcranial Doppler studies. Stroke. 2010;41(11):2697 doi: 10.1161/STROKEAHA.110.594168 20930158 10.1161/STROKEAHA.110.594168

[7] Reinhard M, Wihler C, Roth M, Harloff A, Niesen WD, Timmer J, et al Cerebral autoregulation dynamics in acute ischemic stroke after rtPA thrombolysis. Cerebrovascular Diseases. 2008;26(2):147–55. doi: 10.1159/000139662 18560218 10.1159/000139662

[8] Reinhard M, Rutsch S, Lambeck J, Wihler C, Czosnyka M, Weiller C, et al Dynamic cerebral autoregulation associates with infarct size and outcome after ischemic stroke. Acta Neurologica Scandinavica. 2012;125(3):156–62. doi: 10.1111/j.1600-0404.2011.01515.x 21470192 10.1111/j.1600-0404.2011.01515.x

[9] Dawson SL, Blake MJ, Panerai RB, Potter JF. Dynamic but not static cerebral autoregulation is impaired in acute ischaemic stroke. Cerebrovascular Diseases. 2000;10(2):126–32. doi: 10.1159/000016041 10686451 10.1159/000016041

[10] Giller CA. The frequency-dependent behavior of cerebral autoregulation. Neurosurgery. 1990;27(3):362 2234328 10.1097/00006123-199009000-00004

[11] Ferrari M, Quaresima V. A brief review on the history of human functional near-infraredspectroscopy (fNIRS) development and fields of application. Neuroimage. 2012;63(2):921 doi: 10.1016/j.neuroimage.2012.03.049 22510258 10.1016/j.neuroimage.2012.03.049

[12] Kainerstorfer JM, Sassaroli A, Tgavalekos KT, Fantini S. Cerebral autoregulation in the microvasculature measured with near-infrared spectroscopy. Journal of Cerebral Blood Flow & Metabolism Official Journal of the International Society of Cerebral Blood Flow & Metabolism. 2015;35(6):959. 10.1038/jcbfm.2015.5 PMC4640259 25669906

[13] Brady KM, Lee JK, Kibler KK, Smielewski P, Czosnyka M, Easley RB, et al Continuous Time-Domain Analysis of Cerebrovascular Autoregulation Using Near-Infrared Spectroscopy. Stroke. 2007;38(10):2818 doi: 10.1161/STROKEAHA.107.485706 17761921 10.1161/STROKEAHA.107.485706 PMC2377358

[14] Steiner LA, Pfister D, Strebel SP, Radolovich D, Smielewski P, Czosnyka M. Near-infrared spectroscopy can monitor dynamic cerebral autoregulation in adults. Neurocritical Care. 2009;10(1):122 doi: 10.1007/s12028-008-9140-5 18807218 10.1007/s12028-008-9140-5

[15] Irani F, Platek SM, Bunce S, Ruocco AC, Chute D. Functional Near Infrared Spectroscopy (fNIRS): An Emerging Neuroimaging Technology with Important Applications for the Study of Brain Disorders. Clinical Neuropsychologist. 2007;21(1):9 doi: 10.1080/13854040600910018 17366276 10.1080/13854040600910018

[16] Reinhard M, Schumacher FK, Rutsch S, Oeinck M, Timmer J, Mader I, et al Spatial mapping of dynamic cerebral autoregulation by multichannel near-infrared spectroscopy in high-grade carotid artery disease. Journal of Biomedical Optics. 2014;19(9):97005 doi: 10.1117/1.JBO.19.9.097005 25253194 10.1117/1.JBO.19.9.097005

[17] Li Z, Zhang M, Cui R, Xin Q, Liqian L, Zhou W, et al Wavelet coherence analysis of prefrontal oxygenation signals in elderly subjects with hypertension. 2014;35(5):777–91. doi: 10.1088/0967-3334/35/5/777 24670282 10.1088/0967-3334/35/5/777

[18] Gao Y, Zhang M, Han Q, Li W, Xin Q, Wang Y, et al Cerebral autoregulation in response to posture change in elderly subjects-assessment by wavelet phase coherence analysis of cerebral tissue oxyhemoglobin concentrations and arterial blood pressure signals. Behavioural Brain Research. 2015;278:330–6. doi: 10.1016/j.bbr.2014.10.019 25453742 10.1016/j.bbr.2014.10.019

[19] Cheng R, Shang Y, Jr HD, Saha SP, Yu G. Noninvasive optical evaluation of spontaneous low frequency oscillations in cerebral hemodynamics. Neuroimage. 2012;62(3):1445–54. doi: 10.1016/j.neuroimage.2012.05.069 22659481 10.1016/j.neuroimage.2012.05.069

[20] Rowley AB, Payne SJ, Tachtsidis I, Ebden MJ, Whiteley JP, Gavaghan DJ, et al Synchronization between arterial blood pressure and cerebral oxyhaemoglobin concentration investigated by wavelet cross-correlation. Physiological Measurement. 2007;28(2):161 doi: 10.1088/0967-3334/28/2/005 17237588 10.1088/0967-3334/28/2/005

[21] Peng T, Ainslie PN, Cotter JD, Murrell C, Thomas K, Williams MJ, et al The effects of age on the spontaneous low-frequency oscillations in cerebral and systemic cardiovascular dynamics. Physiological Measurement. 2008;29(9):1055–69. doi: 10.1088/0967-3334/29/9/005 18756026 10.1088/0967-3334/29/9/005

[22] Aneta Stefanovska. Physics of the human cardiovascular system. Contemporary Physics. 1999;40(1):31–55.

[23] Panerai RB, Dawson SL, Potter JF. Linear and nonlinear analysis of human dynamic cerebral autoregulation. Am J Physiol. 1999;277(2):1089–99. 10.1152/ajpheart.1999.277.3.H1089 10484432

[24] Panerai RB. Assessment of cerebral pressure autoregulation in humans—a review of measurement methods. Physiological Measurement. 1998;19(3):305 9735883 10.1088/0967-3334/19/3/001

[25] Papademetriou MD, Tachtsidis I, Elliot MJ, Hoskote A, Elwell CE. Multichannel near infrared spectroscopy indicates regional variations in cerebral autoregulation in infants supported on extracorporeal membrane oxygenation. Journal of Biomedical Optics. 2012;17(6):067008 doi: 10.1117/1.JBO.17.6.067008 22734786 10.1117/1.JBO.17.6.067008

[26] Latka M, Turalska M, Glaubic-Latka M, Kolodziej W, Latka D, West BJ. Phase dynamics in cerebral autoregulation. American Journal of Physiology Heart & Circulatory Physiology. 2005;289(5):H2272. 16024579 10.1152/ajpheart.01307.2004

[27] Stankovski T, Ticcinelli V, Mcclintock PVE, Stefanovska A. Coupling functions in networks of oscillators. New Journal of Physics. 2015;17(3):35002–13(12).

[28] Kralemann B, Pikovsky A, Rosenblum M, Kenner T, Schaefer J, Moser M. In vivo cardiac phase response curve elucidates human respiratory heart rate variability. Nature Communications. 2013;4(4):2418. 10.1038/ncomms3418 23995013

[29] Stankovski T, Duggento A, Mcclintock PV, Stefanovska A. Inference of time-evolving coupled dynamical systems in the presence of noise. Physical Review Letters. 2012;109(2):1–6. 10.1103/PhysRevLett.109.024101 23030162

[30] Von Toussaint U. Bayesian inference in physics. Review of Modern Physics. 2011;83(3):943–99.

[31] Daunizeau J, Friston KJ, Kiebel SJ. Variational Bayesian identification and prediction of stochastic nonlinear dynamic causal models. Physica D Nonlinear Phenomena. 2009;238(21):2089–118. doi: 10.1016/j.physd.2009.08.002 19862351 10.1016/j.physd.2009.08.002 PMC2767160

[32] Penny WD, Litvak V, Fuentemilla L, Duzel E, Friston K. Dynamic Causal Models for phase coupling. Journal of Neuroscience Methods. 2009;183(1):19–30. doi: 10.1016/j.jneumeth.2009.06.029 19576931 10.1016/j.jneumeth.2009.06.029 PMC2751835

[33] Smelyanskiy VN, Luchinsky DG, Stefanovska A, Mcclintock PVE. Inference of a Nonlinear Stochastic Model of the Cardiorespiratory Interaction. Physical Review Letters. 2005;94(9):098101 doi: 10.1103/PhysRevLett.94.098101 15784004 10.1103/PhysRevLett.94.098101

[34] Ticcinelli V, Stankovski T, Iatsenko D, Bernjak A, Bradbury AE, Gallagher AR, et al Coherence and Coupling Functions Reveal Microvascular Impairment in Treated Hypertension. Frontiers in Physiology. 2017;8:749 doi: 10.3389/fphys.2017.00749 29081750 10.3389/fphys.2017.00749 PMC5645539

[35] Tomislav S, Spase P, Johan R, Smith AF, Mcclintock PVE, Aneta S. Alterations in the coupling functions between cortical and cardio-respiratory oscillations due to anaesthesia with propofol and sevoflurane. Philosophical Transactions. 2016;374(2067). 10.1098/rsta.2015.0186 PMC4822446 27045000

[36] Miller EK, Cohen JD. An integrative theory of prefrontal cortex function. Annual Review of Neuroscience. 2001;24(1):167. 10.1146/annurev.neuro.24.1.167 11283309

[37] Basso MS, Bisconti S, Muthalib M, Spezialetti M, Cutini S, Ferrari M, et al A semi-immersive virtual reality incremental swing balance task activates prefrontal cortex: a functional near-infrared spectroscopy study. Neuroimage. 2014;85(2):451–60. 23684867 10.1016/j.neuroimage.2013.05.031

[38] Mihara M, Miyai I, Hatakenaka M, Kubota K, Sakoda S. Role of the prefrontal cortex in human balance control. Neuroimage. 2008;43(2):329–36. doi: 10.1016/j.neuroimage.2008.07.029 18718542 10.1016/j.neuroimage.2008.07.029

[39] Hogenhout M. The age-related regulation of sensorimotor integration in human postural control. Molecular & Cellular Biology. 2013;8(11):4889–95.

[40] Moran J, Desimone R. Selective Attention Gates Visual Processing in the Extrastriate Cortex. Science. 1985;229(4715):782–4. 4023713 10.1126/science.4023713

[41] Corbetta M, Miezin FM, Dobmeyer S, Shulman GL, Petersen SE. Selective and divided attention during visual discriminations of shape, color, and speed: functional anatomy by positron emission tomography. Journal of Neuroscience the Official Journal of the Society for Neuroscience. 1991;11(8):2383. 10.1523/JNEUROSCI.11-08-02383.1991 PMC6575512 1869921

[42] Astafiev SV, Stanley CM, Shulman GL, Corbetta M. Extrastriate body area in human occipital cortex responds to the performance of motor actions. Nature Neuroscience. 2004;7(5):542 doi: 10.1038/nn1241 15107859 10.1038/nn1241

[43] Cope M, Delpy DT. System for long-term measurement of cerebral blood and tissue oxygenation on newborn infants by near infra-red transillumination. Medical & Biological Engineering & Computing. 1988;26(3):289. 2855531 10.1007/BF02447083

[44] Tan Q, Zhang M, Wang Y, Zhang M, Wang Y, Xin Q, et al Frequency‐specific functional connectivity revealed by wavelet‐based coherence analysis in elderly subjects with cerebral infarction using NIRS method. 2015;42(9):5391 doi: 10.1118/1.4928672 26328988 10.1118/1.4928672

[45] Li Z, Wang Y, Li Y, Wang Y, Li J, Zhang L. Wavelet analysis of cerebral oxygenation signal measured by near infrared spectroscopy in subjects with cerebral infarction. Microvascular Research. 2010;80(1):142 doi: 10.1016/j.mvr.2010.02.004 20156461 10.1016/j.mvr.2010.02.004

[46] Townsend NW, Germuska RB. Locating features in a photoplethysmograph signal. WO; 2003.

[47] Xu G, Zhang M, Wang Y, Liu Z, Huo C, Li Z, et al Functional connectivity analysis of distracted drivers based on the wavelet phase coherence of functional near-infrared spectroscopy signals. Plos One. 2017;12(11):e0188329 doi: 10.1371/journal.pone.0188329 29176895 10.1371/journal.pone.0188329 PMC5703451

[48] Scholkmann F, Spichtig S, Muehlemann T, Wolf M. How to detect and reduce movement artifacts in near-infrared imaging using moving standard deviation and spline interpolation. Physiological Measurement. 2010;31(5):649 doi: 10.1088/0967-3334/31/5/004 20308772 10.1088/0967-3334/31/5/004

[49] Li Z, Zhang M, Xin Q, Luo S, Zhou W, Cui R, et al Assessment of cerebral oxygenation oscillations in subjects with hypertension. Microvascular Research. 2013;88(4):32–41. 23583904 10.1016/j.mvr.2013.04.003

[50] Han Q, Zhang M, Li W, Gao Y, Xin Q, Wang Y, et al Wavelet coherence analysis of prefrontal tissue oxyhaemoglobin signals as measured using near-infrared spectroscopy in elderly subjects with cerebral infarction. Microvascular Research. 2014;95(1):108. 25117487 10.1016/j.mvr.2014.08.001

[51] Mertins A, Mertins DA. Signal Analysis: Wavelets, Filter Banks, Time-Frequency Transforms and Applications: John Wiley & Sons, Inc; 1999.

[52] Bandrivskyy A, Bernjak A, Mcclintock P, Stefanovska A. Wavelet Phase Coherence Analysis: Application to Skin Temperature and Blood Flow. Cardiovascular Engineering An International Journal. 2004;4(1):89–93.

[53] Bernjak A, Stefanovska A, Mcclintock Pve, Owen-Lynch PJ, Clarkson Pbm. Coherence between fluctuations in blood flow and oxygen saturation. Fluctuation & Noise Letters. 2012;11(01):1240013-.

[54] Kurths J, Pikovsky A, Rosenblum M. Synchronization: a universal concept in nonlinear sciences. Physics Today. 2002;70(1):47-.

[55] Schwabedal JT, Pikovsky A. Effective phase dynamics of noise-induced oscillations in excitable systems. Physical Review E Statistical Nonlinear & Soft Matter Physics. 2010;81(2):046218. 10.1103/PhysRevE.81.046218 20481818

[56] Stankovski T, Duggento A, Mcclintock PVE, Stefanovska A. A tutorial on time-evolving dynamical Bayesian inference. European Physical Journal Special Topics. 2014;223(13):2685–703.

[57] Schreiber T, Schmitz A. Surrogate time series. Physica D Nonlinear Phenomena. 2000;142(3–4):346–82.

[58] Panerai RB. Transcranial Doppler for evaluation of cerebral autoregulation. Clinical Autonomic Research. 2009;19(4):197–211. doi: 10.1007/s10286-009-0011-8 19370374 10.1007/s10286-009-0011-8

[59] Panerai RB. Cerebral Autoregulation: From Models to Clinical Applications. Cardiovascular Engineering. 2008;8(1):42 doi: 10.1007/s10558-007-9044-6 18041584 10.1007/s10558-007-9044-6

[60] Shiogai Y., Stefanovska A., & Mcclintock P. V. Nonlinear dynamics of cardiovascular ageing. Physics reports. 2010;488(2–3):51 doi: 10.1016/j.physrep.2009.12.003 20396667 10.1016/j.physrep.2009.12.003 PMC2853263

[61] Willie C. K., Tzeng Y. C., Fisher J. A., & Ainslie P. N. Integrative regulation of human brain blood flow. The Journal of physiology. 2014;592(5):841 doi: 10.1113/jphysiol.2013.268953 24396059 10.1113/jphysiol.2013.268953 PMC3948549

[62] Wollner L, Mccarthy ST, Soper ND, Macy DJ. Failure of cerebral autoregulation as a cause of brain dysfunction in the elderly. British Medical Journal. 1979;1(6171):1117–8. 444957 10.1136/bmj.1.6171.1117 PMC1598784

[63] Folkow B. Description of the myogenic hypothesis. Circ Res. 1964;15(1):279–87. 14206315

[64] Kvernmo HD, Stefanovska A, Bracic M, Kirkebøen KA, Kvernebo K. Spectral analysis of the laser Doppler perfusion signal in human skin before and after exercise. Microvascular Research. 1998;56(3):173–82. doi: 10.1006/mvre.1998.2108 9828155 10.1006/mvre.1998.2108

[65] Meyer JU, Borgström P, Lindbom L, Intaglietta M. Vasomotion patterns in skeletal muscle arterioles during changes in arterial pressure. Microvascular Research. 1988;35(2):193–203. 3367792 10.1016/0026-2862(88)90062-3

[66] Johnson PC. The Myogenic Response: John Wiley & Sons, Inc.; 1991. 159–68 p.

[67] Aalkjaer C, Nilsson H. Vasomotion: cellular background for the oscillator and for the synchronization of smooth muscle cells. British Journal of Pharmacology. 2005;144(5):605 doi: 10.1038/sj.bjp.0706084 15678091 10.1038/sj.bjp.0706084 PMC1576043

[68] Katura T, Tanaka N, Obata A, Sato H, Maki A. Quantitative evaluation of interrelations between spontaneous low-frequency oscillations in cerebral hemodynamics and systemic cardiovascular dynamics. Neuroimage. 2006;31(4):1592–600. doi: 10.1016/j.neuroimage.2006.02.010 16549367 10.1016/j.neuroimage.2006.02.010

[69] Addison PS. A Review of Wavelet Transform Time–Frequency Methods for NIRS-Based Analysis of Cerebral Autoregulation. IEEE Reviews in Biomedical Engineering. 2015;8:78–85. doi: 10.1109/RBME.2015.2436978 26011892 10.1109/RBME.2015.2436978

[70] Laurent S, Boutouyrie P, Asmar R, Gautier I, Laloux B, Guize L, et al Aortic stiffness is an independent predictor of all-cause and cardiovascular mortality in hypertensive patients. Hypertension. 2001;37(5):1236–41. 11358934 10.1161/01.hyp.37.5.1236

[71] Terborg C, Gora F, Weiller C, Röther J. Reduced Vasomotor Reactivity in Cerebral Microangiopathy A Study With Near-Infrared Spectroscopy and Transcranial Doppler Sonography. Stroke; a journal of cerebral circulation. 2000;31(4):924–9. 10.1161/01.str.31.4.924 10754000

[72] Bellapart J, Fraser JF. Transcranial Doppler assessment of cerebral autoregulation. Ultrasound in Medicine & Biology. 2009;35(6):883–93. 19329245 10.1016/j.ultrasmedbio.2009.01.005

[73] Zhang R, Witkowski S, Fu Q, Claassen JA, Levine BD. Cerebral hemodynamics after short- and long-term reduction in blood pressure in mild and moderate hypertension. Hypertension. 2007;49(5):1149 doi: 10.1161/HYPERTENSIONAHA.106.084939 17353511 10.1161/HYPERTENSIONAHA.106.084939

[74] Levine BD, Giller CA, Lane LD, Buckey JC, Blomqvist CG. Cerebral versus systemic hemodynamics during graded orthostatic stress in humans. Circulation. 1994;90(1):298–306. 8026012 10.1161/01.cir.90.1.298

[75] Eames PJ, Blake MJ, Dawson SL, Panerai RB, Potter JF. Dynamic cerebral autoregulation and beat to beat blood pressure control are impaired in acute ischaemic stroke. Journal of Neurology Neurosurgery & Psychiatry. 2002;72(4):467. 10.1136/jnnp.72.4.467 PMC1737824 11909905

[76] Schwarz S, Georgiadis D, Aschoff A, Schwab S. Effects of induced hypertension on intracranial pressure and flow velocities of the middle cerebral arteries in patients with large hemispheric stroke. Stroke; a journal of cerebral circulation. 2002;33(4):998. 10.1161/01.str.0000014584.17714.2e 11935051

[77] Schwarz S, Georgiadis D, Aschoff A, Schwab S. Effects of body position on intracranial pressure and cerebral perfusion in patients with large hemispheric stroke. Stroke. 2002;33(2):497–501. 11823659 10.1161/hs0202.102376

[78] Haynes W. Bonferroni Correction: Springer New York; 2013. 154- p.

[79] Ferrari M, Quaresima V. A brief review on the history of human functional near-infrared spectroscopy (fNIRS) development and fields of application. Neuroimage. 2012;63(2):921–35. doi: 10.1016/j.neuroimage.2012.03.049 22510258 10.1016/j.neuroimage.2012.03.049

[80] Tong Y, Frederick BD. Time lag dependent multimodal processing of concurrent fMRI and near-infrared spectroscopy (NIRS) data suggests a global circulatory origin for low-frequency oscillation signals in human brain. Neuroimage. 2010;53(2):553–64. doi: 10.1016/j.neuroimage.2010.06.049 20600975 10.1016/j.neuroimage.2010.06.049 PMC3133965

[81] Gagnon L, Perdue K, Greve DN, Goldenholz D, Kaskhedikar G, Boas DA. Improved recovery of the hemodynamic response in Diffuse Optical Imaging using short optode separations and state-space modeling. Neuroimage. 2011;56(3):1362 doi: 10.1016/j.neuroimage.2011.03.001 21385616 10.1016/j.neuroimage.2011.03.001 PMC3085546

[82] Sasai S, Homae F, Watanabe H, Sasaki AT, Tanabe HC, Sadato N, et al A NIRS-fMRI study of resting state network. Neuroimage. 2012;63(1):179–93. doi: 10.1016/j.neuroimage.2012.06.011 22713670 10.1016/j.neuroimage.2012.06.011

[83] T T, Takikawa Y, Kawagoe R, Shibuya S, Iwano T, Kitazawa S. Influence of skin blood flow on near-infrared spectroscopy signals measured on the forehead during a verbal fluency task. Neuroimage. 2011;57(3):991–1002. doi: 10.1016/j.neuroimage.2011.05.012 21600294 10.1016/j.neuroimage.2011.05.012

[84] Zhang Q, Brown E, Strangman G. Adaptive filtering to reduce global interference in evoked brain activity detection: a human subject case study. Journal of Biomedical Optics. 2007;12(6):064009 doi: 10.1117/1.2804706 18163825 10.1117/1.2804706

[85] Medvedev A, Kainerstorfer J, Sv Barbour R, Vanmeter J. Event-related fast optical signal in a rapid object recognition task: Improving detection by the independent component analysis. Brain Research. 2008;1236(43):145–58. 18725213 10.1016/j.brainres.2008.07.122 PMC2668610

