# Supplementary material for: Alterations in the coupling functions between cerebral oxyhaemoglobin and arterial blood pressure signals in post-stroke subjects
Source: PLoS One. 2018 Apr 18;13(4):e0195936. doi: 10.1371/journal.pone.0195936 (PMC5905974; doi:10.1371/journal.pone.0195936)
Supplement: S1 Table — (PDF) [file pone.0195936.s001.pdf]

S1 Table

Frequency interval	L–H group			R–H group		
	Affected-side	Unaffected-side	p	Affected-side	Unaffected-side	p
Frequency Interval I						
PFC	0.29(0.25)	0.27(0.18)	0.714	0.30(0.18)	0.23(0.15)	0.174
PL	0.27(0.13)	0.29(0.14)	0.119	0.35(0.05)	0.36(0.07)	0.868
OL	0.32(0.14)	0.36(0.10)	0.164	0.36(0.03)	0.40(0.07)	0.079
Frequency Interval II						
PFC	0.19(0.05)	0.19(0.04)	0.579	0.19(0.06)	0.17(0.05)	0.425
PL	0.20(0.05)	0.20(0.05)	0.841	0.20(0.03)	0.19(0.02)	0.451
OL	0.19(0.03)	0.20(0.02)	0.080	0.19(0.03)	0.19(0.03)	0.441
Frequency Interval III						
PFC	0.06(0.01)	0.07(0.01)	0.095	0.07(0.015)	0.09(0.02)	0.075
PL	0.08(0.007)	0.07(0.006)	0.016	0.08(0.008)	0.09(0.02)	0.629
OL	0.08(0.01)	0.08(0.009)	0.397	0.08(0.015)	0.08(0.01)	0.750
Frequency Interval IV						
PFC	0.53(0.61)	0.55(0.54)	0.786	0.62(1.02)	0..72(0.54)	0.740
PL	0.52(0.25)	0.59(0.29)	0.434	0.68(0.37)	0.79(0.53)	0.280
OL	0.34(0.32)	0.50(0.41)	0.186	0.62(0.74)	0.64(0.66)	0.887

Data are presented as mean (SD); *p* values are for the differences between affected side and unaffected side; the *p* values with black fonts indicated the difference was statistically significant.
